# Curcumin-Based Photosensitization, a Green Treatment in Inactivating *Aspergillus flavus* Spores in Peanuts

**DOI:** 10.3390/foods11030354

**Published:** 2022-01-26

**Authors:** Nalukui Mukubesa, Rafael Nguenha, Hung T. Hong, Maral Seididamyeh, Michael E. Netzel, Yasmina Sultanbawa

**Affiliations:** 1 The Ministry of Agriculture, Mulungushi House, Independence Avenue, Lusaka P.O. Box 50197, Zambia; nalukuimukubesa@gmail.com; 2 School of Agriculture and Food Science, The University of Queensland, St. Lucia, QLD 4108, Australia; rafaelnguenha@gmail.com; 3 Faculdade de Agronomia e Engenharia Florestal, Universidade Eduardo Mondlane, Maputo 1102, Mozambique; 4 ARC Industrial Transformation Training Centre for Uniquely Australian Foods, Queensland Alliance for Agriculture and Food Innovation, The University of Queensland, Indooroopilly, QLD 4068, Australia; h.trieu@uq.edu.au (H.T.H.); s.maral@uq.edu.au (M.S.); m.netzel@uq.edu.au (M.E.N.)

**Keywords:** Aflatoxin B1, curcumin, fungal growth, peanuts shelf-life, microbial photoinactivation

## Abstract

Controlling microbial contamination in foods using effective clean and green technologies is important in producing food with less contaminants. This study investigates the effect of photosensitization treatment using naturally occurring curcumin on inactivating *Aspergillus flavus* spores on peanuts. Light dosages of 76.4 J/cm^2^ and 114.5 J/cm^2^ at 420 nm were employed in combination with curcumin concentrations from 25 to 100 μM. The inactivation efficiency of the treatment towards spores in suspension achieved a maximum 2 log CFU/mL reduction in viable spores with 75 μM of curcumin at a light dosage of 114.5 J/cm^2^ (*p* < 0.05). The in vivo study was then designed using the optimum conditions from the in vitro experiment. The photosensitization treatment at three different curcumin concentrations (50, 75, 100 μM) extended the shelf-life of raw peanuts by 7 days when treated with 75 μM of curcumin combined with a 114.5 J/cm^2^ light dosage and stored at 25 °C. The treatment effectively reduced average levels of aflatoxin B1 (AF-B1) on peanuts stored for 7 days at 25 °C from 9.65 mg/kg of untreated samples to 0.007 and 0.006 mg/kg for 75 and 100 μM curcumin (*p* < 0.05) respectively. The results show the potential use of curcumin-based photosensitization treatment in inactivating fungal growth and reducing AF-B1 concentration on raw peanuts.

## 1. Introduction

Peanuts (*Arachis hypogea* L.), also commonly known as groundnuts, are an important food crop due to their nutritional and economic value. Peanuts are rich in proteins, carbohydrates, lipids including monounsaturated and polyunsaturated fatty acids, vitamins and minerals [1,2]. However, the high nutrient content makes peanuts good growth media for the fungus *Aspergillus flavus* [3,4]. The *A. flavus* spores are ubiquitous in nature, mainly contaminating legumes and cereals during production, harvesting, processing and storage [5,6]. The fungi produces aflatoxin B1 (AF-B1), known to be the most potent natural carcinogen [7,8]. Ingestion of foods contaminated with AF-B1 over a period of time causes the aflatoxins to accumulate in the main organs, particularly the liver, causing a condition known as aflatoxicosis which has also been associated with malnutrition in children [9,10].

Several countries have imposed strict regulations for the presence of aflatoxin in food and feed, with limits ranging from 0 to 35 µg/kg [11]. The European Union has the strictest regulations regarding the presence of aflatoxins in food, with maximum acceptable limits of AF-B1 and total aflatoxins of 2 µg/kg and 4 µg/kg, respectively [12,13]. On the other hand, the acceptable limit of total aflatoxins in the United States of America is 20 µg/kg in any product intended for human consumption [13]. In Australia, the acceptable maximum level of total aflatoxins in peanuts is of 15 µg/kg when intended for human consumption [14].

Photosensitization treatment can be used as an alternative to curb fungal growth on peanuts. The treatment combines a photosensitizer and light at the appropriate wavelength, in the presence of oxygen, to produce a cytotoxic effect towards microorganisms [15,16]. The increasing popularity of the photosensitization treatment is due to its cytotoxic effect towards a wide range of microorganisms, including Gram-negative and Gram-positive bacteria, fungi, parasites and viruses, as well as ensuring the non-emergence of resistant strains [17].

The use of natural photosensitizers such as curcumin, a polyphenolic compound predominant in turmeric (*Curcuma longa* L.), is considered a clean and green technology that does not produce toxic chemical residues in food [15,17]. Previous studies of a combination of curcumin and light at an appropriate wavelength, also called curcumin-based photosensitization, showed potential in addressing fungal contamination in foods such as apples [18], dates [19], and maize kernels and flour [20,21,22]. Temba et al. (2016) attained a 2-log reduction of *A. flavus* populations in maize kernels and flour treated with curcumin-based photosensitization [20]. The same authors demonstrated that curcumin-based photosensitization could reduce the accumulation of AF-B1 in maize kernels by over 70% after 10 days of storage at 25 °C, compared to untreated kernels [23]. In another study by Nguenha et al., curcumin-based photosensitization resulted in complete inactivation of *A. flavus* spores in three maize varieties and inhibited the accumulation of AF-B1 after 14 days of storage at 25 °C, while non-treated samples showed a 60-fold increase in the toxin concentration along the same storage period [21]. In fresh produce, photosensitization mediated by curcumin has also shown promising results. Photosensitization mediated by curcumin derivative resulted in over 3 log CFU (Colony Forming Units) reduction in *Escherichia coli* on slices of cucumber, tomato and lettuce using dye concentrations ranging from 10 to 100 µM, combined with a light dose of 33.8 J/cm^2^ [24]. These results show the potential use of curcumin-mediated photosensitization for microbial inactivation in food, which could rule out the use of chemicals and fumigants as one of the interventions commonly used to control spore contamination [19].

Studies recommend that treatments to reduce fungal contamination and aflatoxin accumulation be cost-effective [25]. Curcumin-based photosensitization has the potential to inactivate several microorganisms in food and feed [26,27]. Although previous studies about curcumin-based photosensitization have been conducted in several food products, to the best of our knowledge, the efficiency of this technique to inactivate microorganisms in peanuts has never been evaluated. It is well-documented that the difference in the food matrix limits the applicability of studies conducted in one product on another [22,24,28], posing the need for studies in different food materials. Therefore, this study investigates the potential of curcumin-based photosensitization process as a green novel-based treatment in inactivating *A. flavus* spores on peanuts by exposing the contaminated peanuts to different levels of light doses and curcumin concentration combinations. This approach tested which combination of light and curcumin concentration were most effective in reducing aflatoxin levels and extending the shelf life of peanuts.

## 2. Materials and Methods

### 2.1. Culture Preparation

*Aspergillus flavus* (ATCC 28862) was cultivated on malt extract agar (MEA; Difco, Detroit, MI, USA), incubated at 25 °C for proper spore growth. Then, spores were harvested by flooding the culture surface with 10–15 milliliter (mL) of sterile phosphate buffer solution (pH 7.4; PBS; Sigma-Aldrich, St Louis, MO, USA) containing 0.1% Tween 80 (BDH Chemicals, Kilsyth, VIC, Australia), followed by gently rubbing the surface using a sterile spreader. The spore suspension was then collected into sterile tubes and this procedure was repeated twice. The collected suspensions were mixed and vortexed for 1 min to break any mycelium fragments, followed by centrifugation at 10,000 rotations per minute (rpm) for 1 min to obtain the spore pellets. The supernatant was decanted and 1 mL of sterile PBS containing 0.1% Tween 80 was added and then vortexed to reconstitute the spore suspension. The spore stock suspension was adjusted to the concentration of 107 Colony Forming Units (CFU)/mL) using peptone water (LP0037, Oxoid, Basingstoke, UK) and stored at −20 °C for further experiments.

### 2.2. Photosensitizer and Light Source

A 2000 micromolar (μM) stock solution of curcumin (Sigma-Aldrich) was prepared in ethanol (99.8%, Fisher Scientific, Loughborough, UK). The stock solution was wrapped in foil, stored in the dark at 4 °C, and used within two weeks. The working concentrations (from 25 to 100 μM) were obtained by diluting with distilled water. A Xenon arc lamp machine (Polilight 500^®^, Rofin Forensic, Dingley, VIC, Australia) was used as the light source for photosensitization treatments. The wavelength was determined within the range of 350–650 nanometers (nm) by measuring the absorbance spectra of four different curcumin concentrations using a spectrophotometric plate reader (Infinite 200, Tecan, Männedorf, Switzerland) at room temperature [20]. The wavelength of 420 nm, corresponding to the curcumin absorption peak, was selected and used throughout the light emission treatments, and the illumination was conducted in a lightproof chamber. The distance between the lamp and sample was adjusted to 3 cm. The irradiance of the 420 nm Xenon lamp was 118.71 milliWatts per square centimeter (mW/cm^2)^ at the surface of the samples. The light dosage was calculated by the following equation [29]:

E = Pt

where E is the light dose in Joules per square centimeter (J/cm^2^), P is the irradiance in Watts per square centimeter (W/cm^2^), and t is illumination time in seconds (s).

### 2.3. Photoinactivation of Fungal Spores in Suspension

Aliquots of spore suspension (10^4^ CFU/mL in peptone water) with the same volumes of varying curcumin concentrations (25–100 μM) were mixed and incubated in Petri-dishes (35 mm × 10 mm) in the dark at 25 °C for 10 min. Afterwards, the mixtures were illuminated for 10 and 15 min under constant stirring to provide an even exposure to light. The photoinactivation effect of the treatment on *A. flavus* spores was evaluated by plating 100 μL of suspension into MEA plates and incubating at 25 °C for 3 days. The surviving spore population was enumerated using a colony counter (Stuart Scientific, Stone, UK) and expressed as log10 CFU/mL. To determine the dark toxicity of curcumin against *A. flavus* spores, samples treated with curcumin but not with light were considered as the curcumin treatment set (P^+^/L^0^). The positive control was treated with light without curcumin (P^0^/L^+^) to determine the effect of light alone on spores. Samples treated with neither curcumin nor light were considered as a negative control (P^0^/L^0^). Three sets of experiments were conducted, and all treatments were conducted in triplicate in each set.

### 2.4. Photodecontamination of Inoculated Peanuts

#### 2.4.1. Sample Preparation

Peanuts were purchased from a local market (Brisbane, QLD, Australia), and the skins were removed before further processing. The skinless peanuts were then transferred into a Schott bottle and sterilized using an autoclave (121 °C, 15 psi, 15 min). The peanuts were then stored at room temperature until further experiments. Sterilized peanuts (100 g) were submerged in 200 mL of spore suspension (10^5^ CFU/mL) for 1 min. The liquid was then decanted, and the kernels were dried in a Petri dish at 25 °C for 5 days (with the lid ajar).

The negative control (non-treated inoculated peanuts) ensured the sterility of the peanuts, and the positive control (inoculated peanuts) determined the viability of the spores on the inoculated peanuts. These were assayed by submerging 1.5 g of peanuts from each sample into 5 mL of sterile PBS; the mixture was then vortexed at 1400 rpm for 30 s. Aliquots (100 μL) of the resulting suspension were then spread on the MEA plates and incubated at 25 °C for 3 days.

#### 2.4.2. Photosensitization Treatment

Three curcumin concentrations (50, 75, and 100 μM) were selected according to the in vitro results showing the highest effectiveness among the studied concentrations. Five peanut kernels were dipped in 3 mL of curcumin solution for 30 s. Peanuts were incubated in the dark for 10 min for the curcumin to attach to the spores on the peanut surface. Thereafter, they were illuminated using the Xenon arc light source at 420 nm for 15 min (P^+^/L^+^). To determine the dark toxicity of curcumin, peanuts were dipped in curcumin solution, followed by incubation without illumination (P^+^/L^0^). Two further controls were also evaluated including untreated (P^0^/L^0^) and only light treated (P^0^/L^+^) peanuts. After the photosensitization treatment, samples were dried at 25 °C for 24 h. Next, 1.5 g of each of the samples were submerged in 5 mL of sterile PBS and vortexed for 30 s at 1400 rpm. Thereafter, aliquots (100 μL) of the obtained suspension were spread on MEA plates. The decontamination effect was determined by log reduction (LR) of CFU before and after treatments, using the following equation:

LR = Log_10_ (A) − Log_10_ (B)

where: A represents the CFU/mL of negative control and B represents the CFU/mL of photosensitized spores.

The impact of curcumin-based photosensitization on the shelf life of inoculated peanuts was determined by treating peanut kernels with varying concentrations of curcumin solution (50, 75 and 100 μM) and then dividing them into two groups, the illuminated and the non-illuminated peanuts. The samples were then stored at 25 °C for 10 days and visual observation of fungal growth was conducted after 7 and 10 days of storage.

#### 2.4.3. Aflatoxin Analysis in Peanuts

Extraction of aflatoxin B1 (AF-B1) was carried out following the method in previous studies [23,30] with some modifications. Briefly, ground peanuts (0.5 g) were homogenized with 2 mL of the extraction solution including acetonitrile/Milli-Q^®^ water/formic acid (79/20/1, *v*/*v*/*v*) for 90 min by using a rotary shaker and then centrifuged for 2 min at 3000 rpm at room temperature (Eppendorf Centrifuge 5804, Eppendorf, Hamburg, Germany). The supernatants (350 μL) were mixed with the same volume of mobile phase solution (79:20:1) and filtered through a 0.22 μm hydrophilic PTFE syringe filter into UHPLC vials for AF-B1 analysis by a Shimadzu ultrahigh performance liquid chromatography—electrospray ionization tandem mass spectrometry (UHPLC- ESI-MS/MS) system (Shimadzu, Kyoto, Japan).

**Figure A1:**
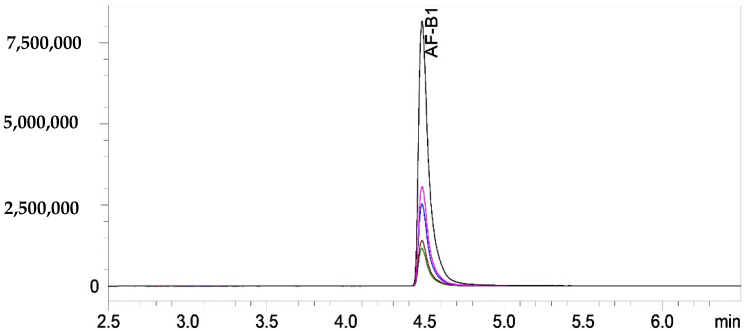
A representative Multiple Reaction Monitoring (MRM) chromatogram of AF-B1 was identified by a Shimadzu UHPLC-MS/MS.

The instrument UHPLC system consists of a system controller (CBM-30A), three pumps (LC-30AD), an autosampler (SIL-30AC), column heater (CTO-20AC), diode-array detectors (DAD) detector (SPD-M30A) and two degassers (DGU-20A3R and DGU-20A5R). The UHPLC system, coupled to a LCMS-8060 triple quadrupole mass spectrometer (Shimadzu) and the ESI source, was operated in positive mode. The multiple Reaction Monitoring (MRM) mode was applied with optimal collision energy (CE) for each fragment ion. The MRM transitions were m/z 313.1 → 285.0 (quantifier) at CE = 20 eV and m/z 341.2 → 269.2 → 241.2 → 214.2 (for qualifier) at 31, 40, and 34 eV, respectively. Chromatographic separation was carried out on an Acquity UPLC HSS T3 column (100 mm × 2.1 mm (i.d.)), 1.8 μm particle size; Waters, Dublin, Ireland), with a run time of 9 min and the column oven at 40 °C. The mobile phase consisted of a gradient program of mobile phase A (MQ-water/formic acid, 99/1 *v*/*v*, and 10 mM ammonium formate and mobile phase B (methanol/water/formic acid, 97/2/1 *v*/*v*/*v*, and 10 mM ammonium formate) at a flow rate of 0.5 mL/min. The elution was programmed with 100% A as the initial isocratic for 2 min; followed by a linear gradient from 100% A to 50% A for 0.5 min, and then 50–0% A for 3.5 min, holding at 0% A for 1.1 min, conditioning for 0.9 min and re-equilibrating for 1 min. Data were collected by using Shimadzu LabSolutions Insight liquid chromatography-mass spectrometry (LC-MS) software version 3.2. The Multiple Reaction Monitoring (MRM) chromatogram from the UHPLC can be found in Appendix A (Figure A1).

### 2.5. Statistical Analysis

All experiments were conducted in three replications. The mean values were statistically compared by ANOVA (one-way analysis of variance) and the Duncan Multiple Range Test using SPSS (version 23; IBM Institute Inc., Armonk, NY, USA). Least significant differences (*p* < 0.05) were used to compare differences between means.

## 3. Results and Discussion

### 3.1. Photoinactivation of Fungal Spores in Suspension

**Figure 1:**
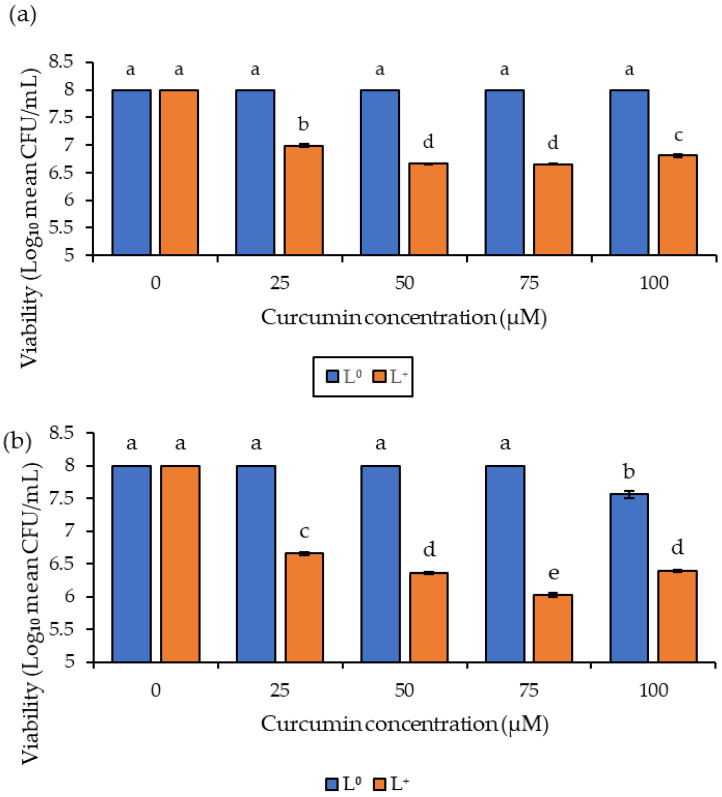
Effect of photosensitization on the viability of *Aspergillus flavus* spores using different curcumin concentrations after light dosage treatment of (**a**) 76.4 J/cm^2^, and (**b**) 114.5 J/cm^2^ at 420 nm. Different letters indicate statistically significant differences (*p* < 0.05) between treatments; n = 3 independent experiments with triplicate samples per condition per experiment.

The in vitro study of the antifungal effect of curcumin-based photosensitization was investigated under different curcumin concentrations (25–100 μM) and light dosages of 76.4 and 114.5 J/cm^2^. The non-treated spores (P^0^/L^0^) showed ~8 log CFU/mL viability, and no significant reduction was observed in samples treated with curcumin without illumination (P^+^/L^0^) or when exposed to light dosages of 76.4 and 114.5 J/cm^2^ illumination without curcumin (P^0^/L^+^) (Figure 1). However, curcumin-based photosensitization (P^+^/L^+^) resulted in a 2 log CFU/mL reduction in spore viability (*p* < 0.05) when treated using a light dosage of 114.5 J/cm^2^ combined with 75 μM of curcumin (Figure 1b), while photosensitization treatment at the lower light dosage of 76.4 J/cm^2^ resulted in a 1.3 log CFU/mL reduction at 50 and 75 μM curcumin concentrations (Figure 1a). These results indicate that the photoinactivation of *A. flavus* spores was dependent on curcumin concentrations from 25 to 75 μM, leading to one more log reduction in spore viability under both light dosages. However, further increases in curcumin concentration gave rise to lower photoinactivation activity. The results also indicated that photoinactivation was light-dosage dependent, becoming more effective with an increase in light dosage. These two observations corroborate with research by Song et al. [18] when inactivating *Penicillium expansum*.

The concentration of the photosensitizer at 75 μM had the most phototoxic effect on the *A. flavus* spores compared to the lower concentrations (25 and 50 μM) and the higher dye concentration (100 μM). Curcumin is known to sparingly dissolve in solution; therefore, the lower the curcumin concentration, the less turbid the solution and the opposite is true for higher curcumin concentrations. The turbidity of the solution affects the degree at which light penetrates the solution and excites the photosensitizer, thereby producing reactive oxygen species (ROS). Therefore, for lower-concentration solutions (25, 50 and 75 μM) light easily penetrates, causing them to be more cytotoxic compared to higher concentrations of curcumin, in this case at 100 μM [19,20]. However, at lower curcumin concentrations, the yield of ROS is not sufficient to effectively reduce spore viability [31,32,33].

### 3.2. Photodecontamination of Inoculated Peanuts

**Figure 2:**
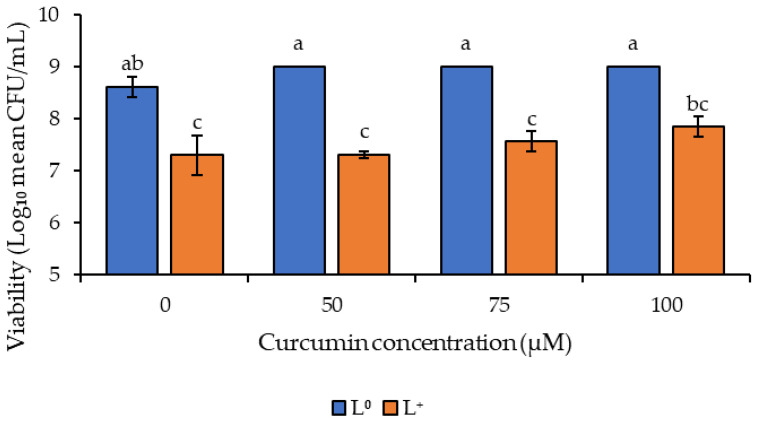
Effect of photosensitization on the viability of *Aspergillus flavus* on peanuts treated in 50, 75 and 100 μM of curcumin using a light dosage of 114.5 J/cm^2^. Different letters indicate statistically significant differences (*p* < 0.05) between treatments; n = 3 independent experiments with triplicate samples per condition per experiment.

This study of the efficiency of curcumin-based photosensitization treatment on whole peanut kernels is the first of its kind. The raw peanuts inoculated with *A. flavus* spores were investigated using three curcumin concentrations (50, 75, and 100 μM) at 114.5 J/cm^2^ illumination (Figure 2). The processing conditions were selected according to the results of the in vitro study. The negative control of non-treated peanuts (P^0^/L^0^) showed an initial spore population of 8.6 log CFU/mL. Curcumin-treated peanuts without illumination (P^+^/L^0^) gave an insignificant change in spore population (*p* > 0.05) (Figure 2). However, light-only treatment (P^0^/L^+^) led to a 1.3 log CFU/mL reduction in spore population, which was significantly different from the negative control (P^0^/L^0^) and photosensitizer only (P^+^/L^0^) treatments (*p* < 0.05) but was not significantly different from the photosensitization treatments at all three concentration levels (*p* > 0.05) which resulted in 1.7, 1.5, and 1.2 log CFU/mL reductions at 50, 75, and 100 of μM curcumin, respectively.

Earlier studies on curcumin-based photoinactivation of *A. flavus* by Temba et al. [20] showed an approximate 2 log CFU/mL reduction in viable spores with 25 and 45 μM of curcumin using 60 J/cm^2^ illumination for both whole maize kernels and milled maize samples. In another study using a curcumin derivative, SACUR-3, as a photosensitizer to inactivate *Escherichia coli*, Glueck et al. [24] achieved over 5 log CFU/mL reduction in fenugreek seeds and only about 1 log CFU/mL reduction in mung beans, which shows the influence of the food matrix in the efficiency of photosensitization treatment. Therefore, comparisons of the above investigative studies may be difficult due to the different experimental environments that include the light source, light intensity, wavelength at which light is emitted, the different microbial strains or species, the concentration of the photosensitizers used and the food matrix. However, in this study, the reduction in viable spores on the peanuts indicates that photoactivated curcumin has the potential to inactivate spore cells.

Studies by Yi et al. [34] reported an increase in the photosensitivity of curcumin in lipophilic environments compared to aqueous environments due to greater solubility in lipids than in water. Due to the high levels of lipids, peanuts are a suitable matrix that can enhance the photosensitization ability of curcumin. Nevertheless, in in vitro studies (using aqueous media) curcumin-based photosensitization registered a higher photoinactivation efficiency with 2 log CFU/mL compared to the photoinactivation on the surface of peanuts (lipophilic medium) where a maximum inactivation of 1.7 log CFU/mL was achieved. Previous studies have also reported lower photoinactivation efficiency on food surfaces than in vitro [28,35], which is attributed to the irregularity of the food and non-uniform distribution of light when a two-dimensional light source is used [24], as is the case in the present study.

The photosensitized peanuts at all three curcumin concentrations showed reduced levels of viable spores but were not significantly different between the three treatments. These results could be attributed to the interaction of other bioactive compounds such as flavonoids, also known to have antioxidative properties [36].

**Figure 3:**
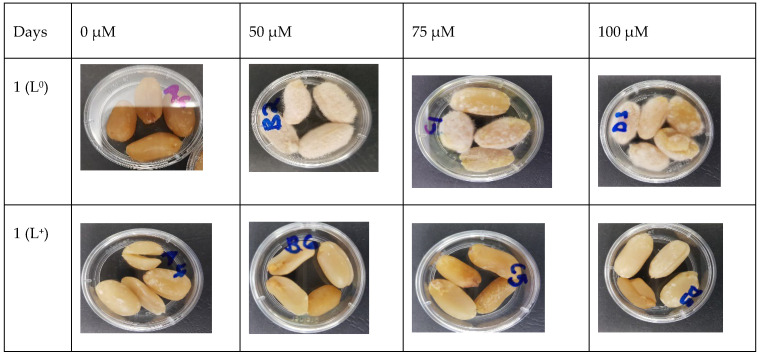

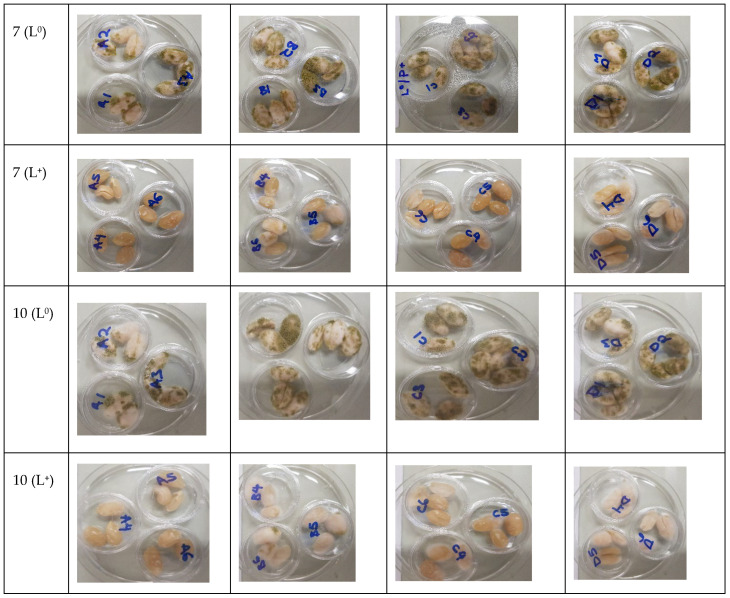
Growth of *Aspergillus flavus* on control and photosensitized peanuts stored at 25 °C for 10 days after the treatment with 114.5 J/cm^2^ of light combined with curcumin (50, 75, 100 µM).

To determine the effect of curcumin-based photosensitization on the shelf life of inoculated peanut kernels, the growth of *A. flavus* on peanut surfaces was visually inspected during 10 days of storage at 25 °C (Figure 3). The light (P^0^/L^+^) and curcumin (P^+^/L^0^) treatments showed visible white, hair-like fungal structures on peanuts from day one; thereafter, there was evident fungal growth on all the untreated peanut samples at 7 days of storage. The curcumin-based, photosensitization-treated peanuts (P^+^/L^+^) with 50 and 100 μM of curcumin only exhibited visible white hair-like structures on the surface of approximately 50% of the peanut kernels on day 7. In contrast, samples photosensitized with 75 μM of curcumin had no visible fungal growth on day 7. A similar trend was observed on day 10 of storage with the curcumin-based photosensitized-treated (P^+^/L^+^) peanuts, which showed an increase in fungal growth and evident white, hair-like structures on peanuts treated with 50 and 100 μM curcumin, respectively, while only white hair-like structures were observed on peanuts treated with 75 μM of curcumin, indicating early signs of visible fungal growth.

The curcumin-based treatment (P^+^/L^+^) was demonstrated to be effective in extending the shelf-life of the inoculated peanut kernels for 7 days at 25 °C, in comparison with the control (P^0^/L^0^), light-only (P^0^/L^+^), and curcumin-only (P^+^/L^0^) treatments. The visual observation of the stored peanuts at 25 °C showed an increase in fungal growth in all samples; however, photosensitized peanuts showed initiation of growth on day 10 of storage. Similar observations were reported by Al-Asmari et al. [19], in whose study curcumin-based photosensitization extended the shelf-life of dates by 14 days using a curcumin concentration of 1400 μM and a light dosage of 180 J/cm^2^. The test on the viability of spores on the peanuts (Figure 2) indicated the effectiveness of the treatment and how the microbial count immediately after the treatment affects the shelf-life quality of the peanuts.

### 3.3. Effect of Curcumin-Based Photosensitization on Aflatoxin B1 Generation

**Figure 4:**
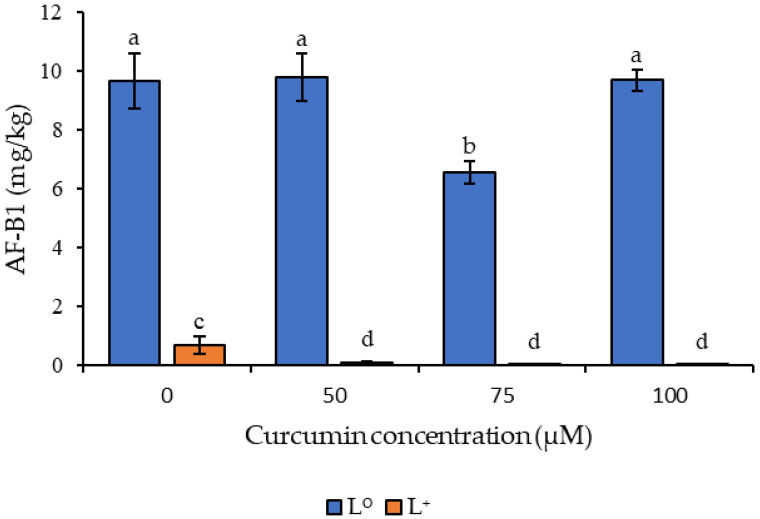
Effect of photosensitization on aflatoxin B1 (AF-B1) generation on *Aspergillus flavus*-inoculated peanuts during storage at 25 °C after 7 days storage. Different letters indicate significant differences (*p* < 0.05) between the treatments; n = 3 independent experiments with triplicate samples per condition per experiment.

A significant effect of the photosensitization treatment on the accumulation of AF-B1 levels on whole peanut kernels stored for 7 days at 25 °C was observed in the current study. The treatment was conducted at three levels of curcumin concentrations (50, 75 and 100 µM) (Figure 4). The control peanuts (P^0^/L^0^) contained 9.65 mg/kg of AF-B1, while the light treatment (P^0^/L^+^) had a reduction in AF-B1 levels, achieving 0.66 mg/kg. The photosensitizer treatment (P^+^/L^0^) at 50, 75 and 100 μM had AF-B1 levels of 9.78, 6.55 and 9.68 mg/kg, respectively. The photosensitization treatment (P^+^/L^+^) at 50, 75 and 100 μM curcumin levels resulted in AF-B1 accumulation of 0.087, 0.0066 and 0.0058 mg/kg, respectively (Figure 4), which were significantly lower (*p* < 0.05) than levels observed in photosensitizer and light treatments.

The results indicated a direct correlation between the spores and levels of aflatoxin B1, with inactivation of the spores resulting in reduced aflatoxin levels, which is in line with previous studies [37]. Photo-inactivated curcumin also acts as an antioxidant, maintaining its cytotoxic properties towards microorganisms through the generation of ROS, triggering apoptosis leading to cell death [34,38], reducing the microbial population, which in turn limits aflatoxin accumulation on food. Interestingly, the photosensitizer treatment (P^+^/L^0^) at 75 μM reduced the level of AF-B1 by 32% compared to the control, resulting in 6.55 mg/kg; this result is significantly different from the other control treatments (*p* < 0.05).

As observed during the in vivo studies on the levels of viable spores, the light-only treatment (P^0^/L^+^) also affected the AF-B1 levels, although the level of AF-B1 in this treatment was still 100-fold higher than that observed in the photosensitized samples using 75 μM or 100 μM of curcumin combined with a light dosage of 114.5 J/cm^2^. Studies involving light-only treatment using UV and LED observed an antimicrobial impact, but at a higher power output or light dosage and, as observed earlier, the light dosage used in this experiment was high and had an impact on the levels of AF-B1 [39,40]. In the light-only treatment (P^0^/L^+^) the peanuts were not dipped in curcumin solution, therefore reducing the moisture content of the samples from 5.3 to 4.2. Reduced moisture content retards the growth of *A. flavus* spores in peanuts, affecting the levels of toxins [41].

## 4. Conclusions

The study reported that curcumin-based photosensitization is a green and potentially effective method of microbial inactivation. The use of this novel technique by combining concentrations of curcumin in the range of 25–75 µM and a light dose of 114.5 J/cm^2^ is able to effectively inactivate and significantly reduce *A. flavus* spores and prolong the shelf-life of peanuts by 7 days. The study has shown that the photosensitization treatment was effective at all three concentrations, significantly reducing the levels of aflatoxin B1 to within the safe limit for human consumption, while the level of the toxin in untreated samples remained high and outside safe limits for consumption. Therefore, this novel technique has a potential use in reducing economic losses by increasing the shelf-life of peanuts during postharvest storage. The use of natural photosensitizers such as curcumin is advantageous to developing countries, as the food additive can be grown and easily worked into the value chain of peanut production and processing. Further studies on the stability of nutritional values under this treatment are still needed. In addition, studies on the metabolic pathway of curcumin during the treatment should be considered.

## Data Availability

The data presented in this study are available on request from the corresponding author.
